# 3D Printing of Concrete-Geopolymer Hybrids

**DOI:** 10.3390/ma15082819

**Published:** 2022-04-12

**Authors:** Celina Ziejewska, Joanna Marczyk, Kinga Korniejenko, Sebastian Bednarz, Piotr Sroczyk, Michał Łach, Janusz Mikuła, Beata Figiela, Magdalena Szechyńska-Hebda, Marek Hebda

**Affiliations:** 1 Faculty of Materials Engineering and Physics, Cracow University of Technology, Warszawska 24, 31-155 Kraków, Poland; celina.ziejewska@gmail.com (C.Z.); jmarczyk94@gmail.com (J.M.); kinga.korniejenko@pk.edu.pl (K.K.); sbednarz39@gmail.com (S.B.); piotrsroczyk22@gmail.com (P.S.); michal.lach@pk.edu.pl (M.Ł.); janusz.mikula@pk.edu.pl (J.M.); beata.figiela@pk.edu.pl (B.F.); 2 Plant Breeding and Acclimatization Institute-National Research Institute, Radzików, 05-870 Błonie, Poland; szechynska@wp.pl

**Keywords:** 3D printing, hybrid materials, concrete, fly ash, metakaolin, fire protection

## Abstract

In recent years, 3D concrete printing technology has been developing dynamically. Intensive research is still being carried out on the composition of the materials dedicated to innovative 3D printing solutions. Here, for the first time, concrete-geopolymer hybrids produced with 3D printing technology and dedicated environmentally friendly building construction are presented. The concrete-geopolymer hybrids consisting of 95% concrete and 5% geopolymer based on fly ash or metakaolin were compared to standard concrete. Moreover, 3D printed samples were compared with the samples of the same composition but prepared by the conventional method of casting into molds. The phase composition, water leachability, compressive, and flexural strength in the parallel and perpendicular directions to the printing direction, and fire resistance followed by compressive strength were evaluated. Concrete-geopolymer hybrids were shown to contain a lower content of hazardous compounds in leaches than concrete samples. The concentration of toxic metals did not exceed the limit values indicated in the Council Decision 2003/33/EC; therefore, the materials were classified as environmentally neutral. The different forms of Si/Al in fly ash and metakaolin resulted in the various potentials for geopolymerization processes, and finally influenced the densification of the hybrids and the potential for immobilization of toxic elements. Although the compressive strength of concrete was approximately 40% higher for cast samples than for 3D printed ones, for the hybrids, the trend was the opposite. The addition of fly ash to concrete resulted in a 20% higher compressive strength compared to an analogous hybrid containing the addition of metakaolin. The compressive strength was 7–10% higher provided the samples were tested in the parallel direction to the *Z*-axis of the printout. The sample compressive strength of 24–43 MPa decreased to 8–19 MPa after the fire resistance tests as a result of moisture evaporation, weight loss, thermal deformation, and crack development. Importantly, the residual compressive strength of the hybrid samples was 1.5- to 2- fold higher than the concrete samples. Therefore, it can be concluded that the addition of geopolymer to the concrete improved the fire resistance of the samples.

## 1. Introduction

Nowadays, concrete is one of the most commonly used substances all over the world, second only to water [1]. Ordinary Portland Cement (OPC) is usually used in concrete production as the binding component [2]. Global industrialization and the huge demand for construction materials have generated an increased interest in cementitious materials [3]. Concrete is the key product in many developing countries, such as China [4], India [5], Nigeria [6], Jordan [7], Turkey [8], and Vietnam [9].

Despite its easy accessibility [10], relatively low cost [11], easy production [12], and other advantages, OPC is unfortunately not free from defects. Some authors found that OPC ordinarily degrades after a certain period of operation and many cracks occur; hence, OPC is brittle and prone to strain and deformation [13]. However, the researchers stated that the concrete harmful environmental impact is definitely more important. The annual greenhouse gas emission caused by the OPC production industry was estimated to be 1.35 billion tons per year, which represents approximately 7% to 9% of the total greenhouse gas emissions [14]. Furthermore, the intensity of carbon dioxide has increased by approximately 31.7% in the past 58 years [11]. There is an urgent need to develop new sustainable materials to avert the worst effects of climate change.

An attractive and more sustainable alternative to OPC is a geopolymer, an inorganic polymer produced by the alkaline activation of silicon-aluminum raw materials, such as red mud, fly ash, metakaolin, slag, rice husk, and glass waste. This reaction is called the polymerization process. Geopolymer production requires lower energy consumption, generates less CO_2_, and can reduce the usage of natural resources and the carbon footprint as compared to OPC [15]. The interest in geopolymer materials is continuously growing due to their low price, excellent durability, good mechanical properties, superb resistance against acids, and elevated temperature [16,17,18,19,20,21]. However, many factors can impact geopolymer material properties [22,23], including: the type and concentration of alkaline activator [24], curing time and conditions [25,26], liquid to solid ratio [27], type of raw materials and their chemical compositions [28,29], mix design [30], SiO_2_/Al_2_O_3_ ratio [31,32],reinforcement [33,34], and the forming process [35,36].

According to their classification [15,37,38], geopolymers are considered a subset of alkali-activated materials (AAM). The geopolymer materials are clustered within the AAM based on the low calcium content, high alkali content, and aluminate content of raw materials (e.g., fly ash [39], slag [40], metakaolin [41]). Thanks to this, the geopolymerization process builds a 3D network with SiO_4_ and AlO_4_ tetrahedrons, cross-linked by bridging oxygen. The alkali cations (K^+^, Na^+^, Ca^2+^, and H_3_O^+^) fill the cavities in these networks, balancing the negative charge of Al^3+^ [42]. However, when the content of Al and alkali (Na and/or K) is lower, calcium-(sodium) aluminosilicate hydrate (C-(N)-A-S-H) gel is primarily forming in the alkali activation process, which has the ability to easily convert into a sodium aluminosilicate hydrate (C-A-S-H) product, depending on the calcium content and the pH value (calcium silicate hydrate does not form the polymerization product) [43].

Additive Manufacturing (AM), commonly known as 3D printing, is a process that enables the production of three-dimensional elements from various materials on small and large scales [44,45]. The 3D printing technique applied in the construction industry provides numerous benefits in comparison to conventional methods. AM produces nearly zero waste, shortens supply and distribution chains, and decreases effects related to the transportation of building materials and elements. Moreover, simplifying construction and customizing products to individuals′ needs gives the possibility of make-to-order components using rapid prototyping methods. Printing on demand reduces the space required to stock inventory. In many cases, AM allows the production of a better performing part; it enables complicated geometries that are desirable but that cannot be made with traditional manufacturing. Taking all of these factors into account, 3D printing technology saves time and costs and is considered to be more sustainable as it has a less harmful impact on the earth and atmosphere [46,47,48,49,50]. Nowadays, it is possible to use various types of materials in the 3D printing process, such as cermets [51], clay [52], metal [53], concrete [54], bio-materials [55], and polymers [56]. Some 3D printed materials have benefits for product performance including being lightweight and possessing a high strength-to-weight ratio, as well as improved heat transfer and energy absorption.

The 3D printing of concrete has become increasingly popular in the construction industry as well as in the research community [57,58]. Numerous studies have focused on the 3D printing of cementitious materials [59,60,61]. However, limited information is available for concrete-geopolymer hybrid products obtained with the 3D printing process, particularly regarding research optimizing the composition of raw materials, their physical and mechanical properties, and the methods of their preparation. Therefore, this work focuses on printed concrete-geopolymer hybrids consisting of 95% concrete and 5% geopolymer based on fly ash or metakaolin. Newly developed hybrids should meet the guidelines for the acceptable ranges of cement content modification, thanks to which they will have the possibility of quick implementation and approval for general use. The analyzed mixtures can be used in the process of printing residential houses via 3D technology, which will enable the development of a universal residential building manufacturing technology, with a structure that is easy to transport and quick to assemble, as well as with the possibility of simple and quick expansion depending on the users′ needs.

Water-leaching tests, mineralogical and mechanical analysis, and fire resistance tests were performed. The 3D printed samples were compared with samples of the same composition that were prepared by the conventional method of casting into molds. Moreover, the effects of printing directions on the mechanical properties of the samples were investigated.

## 2. Materials and Methods

### 2.1. Materials

In this study, OPC, fly ash, metakaolin, and fine aggregate river sand were used as the starting raw materials.

Górażdże Cement S.A. (Heidelberg Cement Group, Chorula, Poland) supplied the commercial cement CEM I 42.5 for this study. The cement used meets the requirement of PN-EN 197-1. The quartz sand was purchased from a local supplier (Świętochłowice, Poland). Fly ash was supplied from the Skawina CHP plant (Skawina, Poland). Based on the chemical composition (Table 1), the fly ash was defined as class F in accordance with ASTM C618 [62]. Metakaolin KM 60 was supplied by Keramost (Kadaň, Czech Republic) (Table 1). The chemical composition, mineralogical characterization, morphology, and thermal and structural properties of all raw materials used in this study were characterized in detail in our previous work [63].

### 2.2. Production of Geopolymer

Fly ash or metakaolin was mixed with sand (1:1) in the GEOLAB cement mortar mixer for 3 min (Geolab, Warsaw, Poland). Alkaline activators were prepared 24 h before geopolymer production. The mixture of alkaline activators consisted of 10 M sodium hydroxide and an aqueous solution of sodium silicate (R-145, ChemiKam, Będzin, Poland) in a ratio of 2.5:1. The mixture was kept at ambient temperature. Then, the alkaline activator was added to the raw materials. The solid to liquid ratio was 0.25 in geopolymers that contained fly ash; and 0.35 in the case of geopolymers based on metakaolin. The components were mixed for 15 min at a speed of approximately 100 rpm.

### 2.3. Production of Concrete

The cement and sand were mixed with a 1:1 ratio. The liquid to solid ratio was 0.125.

### 2.4. Production of Hybrid Materials

To prepare the concrete-geopolymer hybrids, the geopolymer and the concrete mixture were prepared separately. Then, both components were mixed for 15 min at a speed of approximately 100 rpm. The obtained blends were formed by means of a 3D printing or casting method. The compositions of the produced samples are shown in Table 2.

For the casting method, the blend was filled into the mold, and rigorous shaking was applied to evenly distribute the blend throughout the mold. Samples were subsequently cured for 24 h at 75 °C and after demolding, the specimens were kept for ambient curing.

In the case of the 3D printing method, the geometry of the sample was designed in the open-source and free 3D computer graphics Blender software. Samples were printed using the ATMAT Galaxy (ATMAT, Kraków, Poland) 3D printer. The quantity of material mixture used in the printing process was 50 kg. The printing speed was 150 mm s^−1^. Layers of 30 mm thickness were printed with a print head diameter of 15 mm.

### 2.5. Methods

The mineralogical investigation and the Rietveld quantitative phase analysis of the concrete and concrete-geopolymer hybrids based on fly ash and metakaolin were determined using X-ray diffraction (XRD). The X-ray diffraction patterns were recorded on a PANalytical Aeris (Malvern Panalytical, Lelyweg 1, Almelo, Netherlands) diffractometer in the scan range from 10° to 80° 2θ using Cu-Kα radiation, with a step size of 0.003° (2θ) and a time per step of 340 s. The High Score Plus software (PANalytical) and the ICDD (International Center for Diffraction Data, PDF4+) crystallographic database were used to identify the XRD diffractograms.

The leaching test was carried out in accordance with the 12457-4:2006 standard. The pH of solutions was determined by potentiometry. The concentration of the Cl^−^ and SO_4_^2−^ was measured with ion chromatography, whereas the content of Hg was measured with the Cold Vapor Atomic Absorption (CVAA) spectroscopy. The concentrations of Zn, Cd, Cu, Pb, Ni, Ba, Cr, As, Se, Mo, and Sb were measured via Inductively Coupled Plasma Optical Emission Spectrometry (ICP-OES). The content of the total dissolved solids (TDS) was analyzed with the gravimetric method and the dissolved organic carbon (DOC) was examined by Fourier Transform Infrared Spectroscopy (FTIR).

Flexural strength tests were performed via a three-point bend test using the concrete press MATEST 3000 kN (Matest, Treviolo, Italy) in accordance with the PN-EN 12390-5:2019 standard. The dimensions of the specimens were 50 mm × 50 mm × 200 mm, and the tests were carried out in the perpendicular and parallel directions relative to the print direction in the same way as for the compression test after 28 days of curing. Measurements for each of the analyzed material variants were performed with at least three repetitions.

**Figure 1:**
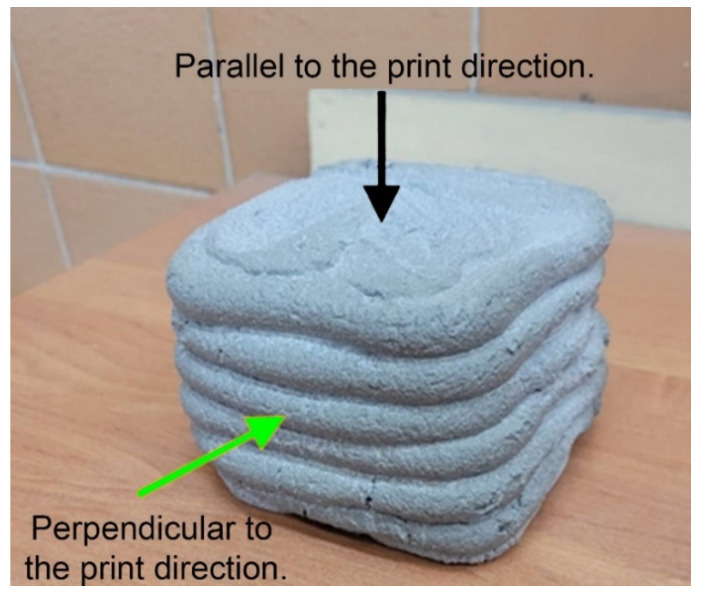
Scheme of applied testing directions for the compressive strength of printed samples.

The compressive strength test of geopolymers was conducted using the concrete press MATEST 3000 kN (Matest, Treviolo, Italy) in accordance with the PN-EN 12390-3:2019 standard. The measurements were carried out on cubic samples 50 mm × 50 mm × 50 mm after 28 days of curing. Moreover, for 3D printed samples, the tests were carried out in two different directions, as shown in Figure 1. Measurements for each of the analyzed variants were performed with at least three repetitions.

Fire-proof resistance testing was carried out based on the PN-EN ISO 1182:2020 standard. The dried samples were set into the preheated electric laboratory oven at 750 °C for a 30 min soaking time. The specimens were cooled down to environmental temperature inside the furnace afterward. The mass loss of the examined samples was measured by weighing the specimens before and after the investigation using a Radwag PS 200/2000R2 (RADWAG Wagi Elektroniczne, Radom, Poland) balance [63]. Measurements for each of the analyzed materials variants were performed with at least three repetitions.

The weight loss for each sample was determined according to the following equation:

(1) $$Loss\;of\;mass,\;\%=100\times \left[1-\frac{mass\;after\;experiment}{mass\;before\;experiment}\right]$$

Moreover, compressive testing was performed on the samples after the fire resistance test. Measurements for each of the analyzed materials variants were performed with at least three repetitions.

## 3. Results

### 3.1. Mineralogical Composition

**Figure 2:**
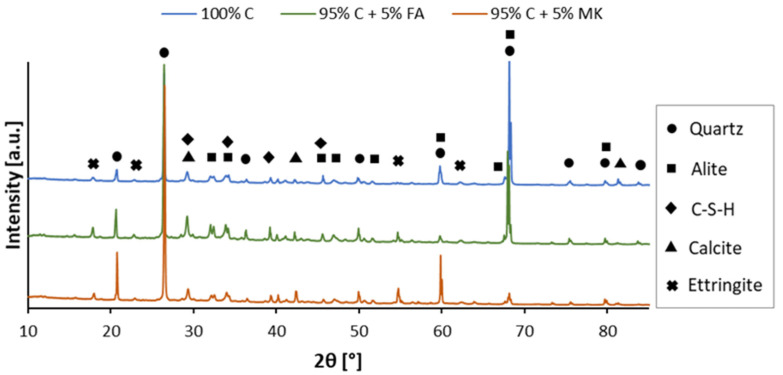
XRD patterns of concrete (100% C) and concrete-geopolymer hybrids based on fly ash (95% C + 5% FA) and metakaolin (95% C + 5% MK).

The qualitative results of XRD (Figure 2) and their quantitative analysis using the Rietveld method (Table 3), performed for both concrete and concrete-geopolymer hybrids, showed the presence of Si-, Al- and Ca-rich phases, such as quartz (SiO_2_, ref. code: 01-089-8938), alite (3CaO·SiO_2_, ref. code: 00-055-0738), calcite (CaCO_3_, ref. code: 00-003-0596), C-S-H in the form of rosenhahnite (Ca_3_Si_3_O_9_·H_2_O, ref. code: 00-019-0250), and ettringite (Ca_6_Al_2_(SO_4_)_3_(OH)_12_·26H_2_O, ref. code: 04-013-3691).

In all samples, the major peaks with the highest intensity were related to quartz; they were found at approximately 25–28° and 68°. The peak at 68° corresponded to alite. The quantitative analysis of the tested samples showed that the quartz content was higher in the samples prepared from hybrid materials. Particularly, quartz content was the highest in the 95% C + 5% FA sample. The different forms of Si/Al suggested the various potentials for geopolymerization processes. The crystalline quartz phase, due to the aluminosilicate compounds, can improve the physical and mechanical properties of geopolymers. Partially replacing the cement with fly ash reduced the clinker phase, i.e., alite. A similar effect was observed in the studies of Jain et al. [64]. Burduhos Nergis et al. [65] observed that samples with a higher Ca content are characterized by a shorter setting time, resulting from the differences in solubility between divalent and trivalent metals. Therefore, the presence of alite is critical for the buildup of strength in the early stages of hydration [63]. Comparing the XRD patterns, the C-S-H gel was found to be higher, provided that the MK-based geopolymer was introduced into the concrete mix; the diffraction peaks and the content of the C-S-H phase increased significantly compared to both the 100% concrete and 95% C + 5% FA samples. A similar relationship was noticed by Liu et al. [66]. In contrast, the calcite and ettringite peaks were the most intense for the 95% C + 5% FA hybrid material. This resulted from the higher Al_2_O_3_ content in the FA than in the cement; the partial replacement of cement with FA could lead to a reduction in the SO_3_/Al_2_O_3_ ratio in the mixture. The formation of ettringite generally promotes the densification of the concrete [64,66] and is crucial for the fast-hardening process. This phase is also described as one of the main phases in cement systems responsible for the immobilization of contaminants and waste in concrete barriers [67].

Comparing the XRD patterns shown in Figure 2 to the XRD patterns of the raw materials [63,68] and geopolymers obtained in previous studies [69,70,71,72,73], it can be concluded that peaks originating from quartz appeared at the same angular positions. In concrete samples and concrete-geopolymer hybrid materials, the peaks derived from kaolinite disappeared from the angular positions of about 12°(2θ) and 24°(2θ). Due to the small content of metakaolin in the 95% C + 5% MK sample, no characteristic hump was observed, which appeared in the XRD spectrum of the metakaolin and MK-based geopolymers. In the concrete-geopolymer hybrids, a distinct peak around 68°(2θ), derived from quartz or alite, appeared. For the geopolymer samples, the peak at this position had a low intensity.

### 3.2. Leaching Tests

The major physico-chemical characteristics of the leachates of concrete and concrete-geopolymer hybrids are presented in Table 4. The pH values of concrete and hybrid leachates were strongly alkaline and ranged from 12.64 to 12.67. The presence of free lime (CaO) and magnesium oxide (MgO) in the material may have increased the pH value. This high pH value was mainly due to the hydrolysis of the lime which produced free hydroxyl ions [74]. Moreover, free lime is limited in cement; therefore, β-dicalcium silicate (C_2_S), and tricalcium silicate (C_3_S) hydration reactions that form portlandite can be also considered.

The total dissolved substances decreased in the following order: concrete-geopolymer hybrids based on fly ash (95% C + 5% FA) > metakaolin (95% C + 5% MK) > concrete (100% C). Analysis revealed the presence of low values of chlorides, fluorides, and sulfates; all of which were most abundant in the concrete. Potentially toxic elements such as Zn, Cu, Pb, Ba, and Cr were present; however, the concentrations of all toxic metals in the tested materials were below the limit values for inert waste in landfills according to Council Decision 2003/33/EC [75]. The remaining metals, such as As, Cu, Cd, Mo, Ni, Se, and Sb, were also released to the leachates in concentrations below the permitted limit values. In most cases, the concrete samples had slightly higher concentrations of the tested elements compared to hybrid samples. Low-Ca geopolymers had an ability to immobilize the ions, due to the cross-linked network, and therefore their leaches had minor contents of tested elements. When comparing the hybrids, 95% C + 5% MK samples had lower concentrations of toxic metals, except for Pb and Zn, which were slightly higher for fly ash-based hybrids. A high concentration of F- ions and an alkaline pH increases the solubility of natural organic substances [76]. The concrete sample was characterized by a higher content of fluorides, and a slightly higher pH, and thus a higher presence of dissolved organic carbon at the level of 6.30 mg/dm^3^ was observed. Dissolved organic carbon is also a parameter important when evaluating the effectiveness of the geopolymerization process. The addition of geopolymers to the concrete reduced the concentration of DOC. In the 95% C + 5% FA sample, the DOC content was found at the level of 5.76 mg/dm^3^. The 95% C + 5% MK sample has the lowest DOC value of 4.76 mg/dm^3^. Although the obtained DOC values were common, thus confirming that FA and MK were suitable raw materials for high-efficiency geopolymerization, it is worth noting that the residual carbon in materials can result from an inefficient coal combustion process (i.e., FA) in the power plant. Generally, the residual carbon can absorb water and chemical admixtures, resulting in a changed air–void system in the material. It can negatively affect the production of building materials and reduce their frost resistance [63].

### 3.3. Flexural Strength

**Figure 3:**
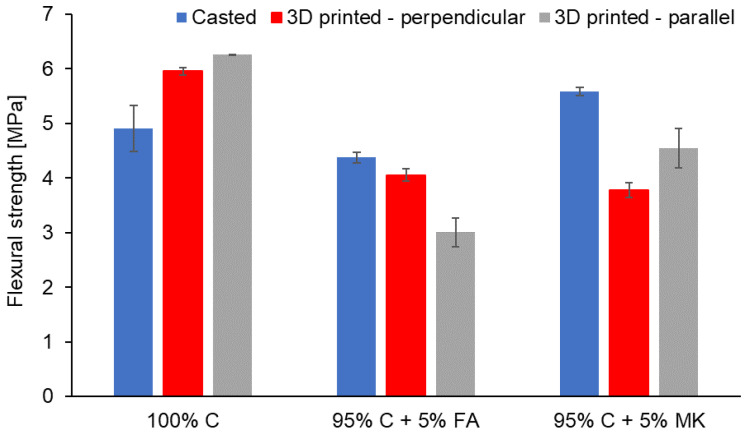
The flexural strength of concrete (100% C) and concrete-geopolymer hybrids based on fly ash (95% C + 5% FA) and metakaolin (95% C + 5% MK) that were cast and 3D printed. For 3D printed samples, the analysis was performed in the parallel and perpendicular direction to the *Z*-axis of the printout.

Figure 3 presents the results of the flexural strength of concrete samples and concrete-geopolymer hybrids produced with conventional and additive methods.

Samples prepared by the conventional method were analyzed in the direction perpendicular to the casting, while, samples prepared with 3D printing technology were analyzed in both directions, i.e., parallel and perpendicular to the *Z*-axis of the printout.

Among the tested samples, a higher flexural strength was recorded for concrete (100% C) when compared to cement with geopolymer additions (95% C + 5% FA and 95% C + 5% MK) (Figure 3). Within concrete samples, slightly higher values of flexural strength were obtained for samples produced in the 3D printing process (5.96 MPa) in relation to the values recorded for samples produced in a conventional way (4.91 MPa). On the basis of the obtained results for conventionally made materials, it was also concluded that hybrids with the addition of MK have a higher strength (5.58 MPa) than hybrids with FA (4.06–4.29 MPa). Similarly, Fuzail Hashmi et al. [77] showed that concrete samples with FA had a lower strength than plain concrete. As the content of FA in the concrete increased, the load-carrying capacity decreased accordingly. This resulted from the cracks, which were wider in FA-concrete hybrids than in conventional concrete. Furthermore, comparing geopolymers, better mechanical properties of metakaolin-based geopolymers were obtained than for FA-based geopolymers [63]. In contrast, according to Adanagouda et al. [78,79], replacing cement with FA or MK in the amount of 10% is optimal for maximal flexural strength development. Chand et al. [80] presented a hybrid cement with MK that achieves a higher flexural strength by 8.29% compared to concrete samples. Testing 3D printed hybrids with FA and MK, the strength of 3D printed samples was found to decrease in comparison to samples produced with the traditional method (Figure 3). Altogether, the presented results showed that the 3D printing process improved the flexural strength of cement while weakening the flexural strength of hybrids. This may be due to the fact that the degree of compaction of the printed sample is lower than that of the sample cast to the mold for which external shaking was used to remove internal air bubbles and evenly distribute the blend throughout the mold [81].

The flexural strength of the 3D printed samples showed anisotropy depending on the direction of sample loading in the parallel or perpendicular direction to the *Z*-axis of the printout (Figure 3). The 3D printed concrete and 95% C + 5% MK hybrids bent parallel showed a flexural strength about 5–16% higher than the samples bent perpendicular to the printing direction. A similar result was found in the studies of Chen et al. [82]. However, Wolfs et al. [83] noticed comparable flexural strength of cast samples and samples bent in the printing direction. The lowest values were obtained for 95% C + 5% FA hybrids (Figure 3). For this type of material, the samples bent parallel showed the lowest flexural strength of 3.01 MPa. The flexural strength along the perpendicular direction was higher than in the parallel direction. Similar results were also observed by Yu et al. [81] and Sanjayan et al. [84]. Researchers have shown that the samples tested in the perpendicular direction had a higher strength than the samples tested in the parallel direction to the *Z*-axis of the printout. It was due to the compression effect of the bottom layer exerted by the gravitational force of the top layers during printing. Therefore, the compaction effect of the bottom area of the 3D printed sample was better. This increased the load capacity of the bottom layer, which resulted in a higher flexural strength [85].

### 3.4. Compressive Strength

**Figure 4:**
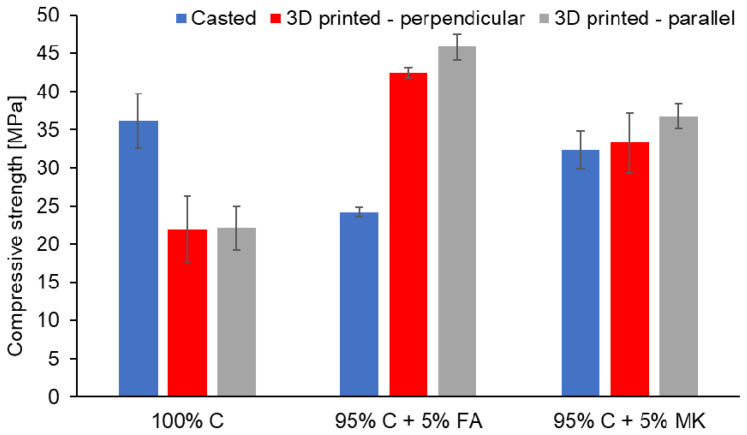
The compressive strength of concrete (100% C) and concrete-geopolymer hybrids based on fly ash (95% C + 5% FA) and metakaolin (95% C + 5% MK) that were cast and 3D printed. For 3D printed samples, the analysis was performed in the parallel and perpendicular direction to the *Z*-axis of the printout.

The results of compressive strength for the samples produced by means of the two methods and under different loading directions for 3D printed samples are presented in Figure 4. It can be observed that the compressive strength of concrete was higher for the samples produced by the casting method compared to 3D printing. These effects were consistent with the results obtained by other authors. It can be concluded that 3D printing caused a decrease in the compressive strength of concrete samples [86,87,88]. However, the addition of geopolymer in the tested material reversed this trend. As can be seen, the compressive strength of the printed hybrids was significantly higher in comparison with the molded samples, regardless of the starting material used for geopolymer synthesis. Moreover, the addition of 5% FA geopolymer resulted in a higher compressive strength in comparison with the addition of 5% MK geopolymer. Regarding the influence of the loading directions on the compressive strength of printed samples, the results show that the compressive strength of the specimens investigated in the parallel direction was higher as compared to the samples tested in the perpendicular direction to the *Z*-axis of the printout. Particularly, in the case of 95% C + 5% FA printed samples, anisotropic behavior was clearly visible. According to the research conducted by other authors, concrete has a better mean compressive strength in the perpendicular direction compared to the parallel direction [89]. However, the compressive strength dependence of the loading directions for the hybrid materials examined in our study was higher in a parallel direction in 95% C + 5% FA samples.

### 3.5. Fire Resistance

**Figure 5:**
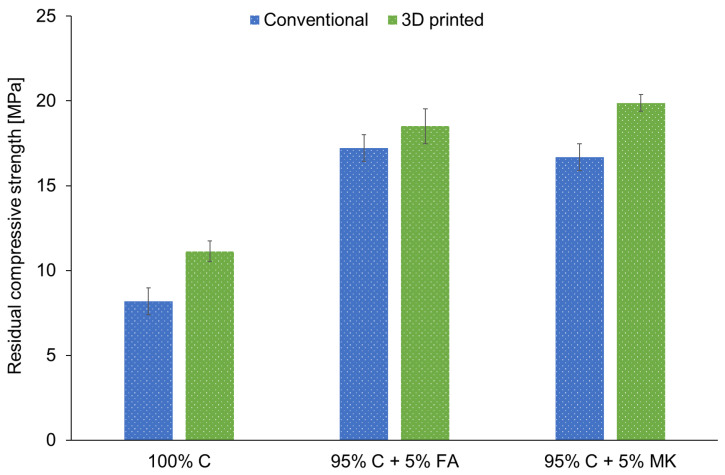
The residual compressive strength after the fire resistance tests for concrete (100% C) and concrete-geopolymer hybrids based on fly ash (95% C + 5% FA) and metakaolin (95% C + 5% MK) that were cast and 3D printed.

Figure 5 shows the results for the residual compressive strength after the fire resistance tests for samples obtained by 3D printing and casting. It was observed that the residual compressive strengths of hybrid samples were significantly higher than for concrete specimens. This result clearly indicates that the addition of geopolymer to cement has improved the fire protection of materials. Moreover, 3D printed concrete and hybrids had a higher residual compressive strength when compared to cast samples. It is well known that the content and type of alkali activator used and the solid/liquid ratio in geopolymer samples influence their chemical structure, mechanical, and thermal properties [90,91]. Metakaolin-based geopolymers required a larger amount of alkaline solution due to their workability, as compared to fly ash-based geopolymers. As a consequence, the hybrid materials with FA had a slightly higher residual compressive strength compared to the samples with MK. The compressive strength of materials after the fire resistance test decreased as a result of the moisture evaporation and spalling of free and bound water inside the samples, which was visible in mass loss and thermal distortion [92,93].

**Figure 6:**
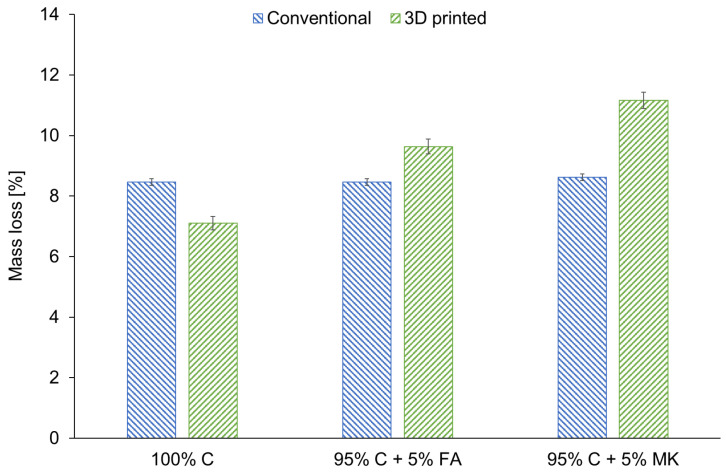
The mass loss after fire-resistance tests for concrete (100% C) and concrete-geopolymer hybrids based on fly ash (95% C + 5% FA) and metakaolin (95% C + 5% MK) that were cast and 3D printed.

Figure 6 depicts the mass loss of samples after exposure to an elevated temperature. Generally, the mass loss of the samples after the fire-resistance test is detrimental in terms of material quality [94,95]. It was observed that the samples produced by casting had a mass loss of approximately 8%, regardless of their composition. However, the 3D printed specimens had higher mass loss after fire-resistance tests in comparison with the conventionally prepared samples. On the basis of these results, it can also be concluded that hybrid materials with FA had lower mass loss than hybrids with MK independently of the manufacturing method used. The mass loss of samples with MK was the most significant among all analyzed samples, probably as a result of the liquid-to-solid ratio in this material. Geopolymer mixtures made on the basis of MK had a liquid-to-solid ratio of 0.35, whereas for FA-containing geopolymers and concrete, the liquid-to-solid ratios were 0.28 and 0.125, respectively. A similar observation was also reported by Kong et al. [96]. They showed that MK-based geopolymers had a significantly higher mass loss than FA-based geopolymers after exposure to elevated temperatures [96]. Altogether, it can be concluded that all the investigated materials are classified as class A1_fl_ in accordance with the PN-EN ISO 1182:2020 standard.

Table 5 shows the surfaces of concrete and concrete-geopolymer hybrids that were cast and 3D printed and exposed to the fire resistance test.

It is well known that concrete structures must provide sufficient fire resistance. The key problems of this building material, however, are cracking and the loss of mechanical strength after exposure to fire. Exposure to high temperatures causes shrinkage or thermal expansion, which in turn causes macrocracks [97]. Indeed, cracks on the surface of the concrete developed after fire resistance tests (Table 5). On the other hand, the surface of the conventionally produced concrete-geopolymer hybrids showed cracks that were smaller in depth and width than in the concrete. These results confirmed the positive effect of the addition of geopolymer to the concrete mixture. Moreover, on the surface of the cement-FA hybrids, there were smaller cracks than for the cement-MK hybrids. This occurred because MK-based geopolymers require more water for workability purposes compared to FA-based geopolymers [97]. It was also observed that the samples produced by the 3D printing process generally showed smaller cracks compared to the cast samples. This effect was related to the fact that the print layers limit the crack propagation process.

## 4. Conclusions

This work presents innovative concrete-geopolymer hybrids, successfully produced by 3D printing technology and dedicated for use as environmentally friendly building materials. The objective of this study was the production of hybrids with an optimal composition i.e., consisting of 95% concrete and 5% geopolymer, which is based on fly ash or metakaolin. Along with the usage of appropriate raw materials, the pre-optimized printing parameters such as the layer thickness, print speed, and print nozzle geometry determined the precise prints. On the basis of the results, it can be concluded that:

1. The chemical analysis of concrete and hybrid leachates showed that the concentration of toxic metals was below the limit values; therefore, the materials can be classified as environmentally neutral. The concentration of toxic metals in the tested samples did not exceed 5% of the permissible values that are indicated in Council Decision 2003/33/EC. In general, the leachate of concrete-geopolymer hybrids had a lower content of hazardous compounds compared to concrete samples. Thus, the addition of geopolymer makes the obtained composites more environmentally friendly than concrete.
2. The compressive strength of concrete was approximately 40% higher for cast samples than for 3D printed ones. However, for hybrids, this trend was reversed. The addition of fly ash to concrete resulted in a 20% higher compressive strength compared to analogous hybrid mixtures containing the addition of metakaolin. Furthermore, the compressive strength of the samples tested in the parallel direction was 7–10% higher compared to the specimens analyzed in the perpendicular direction to the *Z*-axis of the printout.
3. The sample compressive strength of 24–43 MPa decreased to 8–19 MPa after fire resistance tests. This resulted from moisture evaporation, weight loss, thermal deformation, and crack development. The residual compressive strengths of all the investigated hybrid samples were 1.5- to 2-fold higher than for the concrete samples. Therefore, it can be stated that the addition of geopolymer to the concrete improved the fire resistance of the samples. The 95% C + 5% FA hybrids had a 4% higher residual compressive strength than the 95% C + 5% MK samples. It was also observed that the samples produced by the 3D printing process showed smaller cracks after fire resistance tests compared to the cast samples. On the basis of the obtained results, it was concluded that all the tested materials can be classified as class A1_fl_.

Future research will concern the use of concrete-geopolymer hybrids for printing on large-format 3D printers. Ultimately, the components produced in this way could be used in the construction industry.

## Figures and Tables

**Table 1:** The chemical composition of the fly ash and metakaolin determined by X-ray fluorescence analysis, wt% [63].

Component	Fly Ash (%)	Metakaolin (%)
SiO_2_	48.220	52.430
Al_2_O_3_	26.130	42.750
Fe_2_O_3_	7.010	1.200
CaO	5.120	0.490
K_2_O	3.480	1.300
Na_2_O	1.615	0.000
MgO	1.720	0.175
SO_3_	1.110	0.030
TiO_2_	1.110	0.310
P_2_O_5_	0.700	0.440
Cl	0.090	0.060

**Table 2:** Designation of samples depending on the used mix proportions per 50 kg of produced material. C—concrete; FA—fly ash; MK—metakaolin; OPC—Ordinary Portland Cement.

Sample	C Mixture		Geopolymer Mixture		
	OPC	Sand	MK	FA	Sand
C	25	25	-	-	-
95% C + 5% FA	23.75	23.75	-	1.25	1.25
95% C + 5% MK	23.75	23.75	1.25	-	1.25

**Table 3:** Quantitative analysis of concrete (100% C) and concrete-geopolymer hybrids based on fly ash (95% C + 5% FA) and metakaolin (95% C + 5% MK).

		Sample		
		100% C	95% C + 5% FA	95% C + 5% MK
	Quartz	56.5	56.8	69.4
				
	Alite	31.2	24.1	19.2
	C-S-H	2.7	7.3	6.8
Phase (%)	Calcite	9.1	10.3	3.4
	Ettringite	0.6	1.5	1.2

**Table 4:** Physico-chemical analysis of water leachates collected from concrete (100% C) and concrete-geopolymer hybrids based on fly ash (95% C + 5% FA) and metakaolin (95% C + 5% MK).

Samples	Unit	100% C	95% C + 5% FA	95% C + 5% MK
pH of the water extract	-	12.67 ± 0.08	12.64 ± 0.08	12.64 ± 0.08
Total dissolved substances	mg/dm^3^	2032 ± 80	2109 ± 83	2098 ± 82
Chlorides		7.52 ± 0.62	6.97 ± 0.58	6.70 ± 0.56
Fluorides		0.5 ± 0.1	0.4 ± 0.1	0.4 ± 0.1
Sulfates		11.5 ± 0.9	5.14 ± 0.42	3.14 ± 0.26
Zn		0.0030 ± 0.0005	<0.001	0.0060 ± 0.0009
Cd		<0.001	<0.001	<0.001
Cu		0.0020 ± 0.0002	<0.001	<0.001
Pb		0.252 ± 0.037	<0.001	0.035 ± 0.005
Ni		<0.001	<0.001	<0.001
Ba		0.765 ± 0.170	0.805 ± 0.179	0.489 ± 0.109
Cr		0.096 ± 0.022	0.063 ± 0.015	0.050 ± 0.012
Cr (VI)		0.090 ± 0.005	0.061 ± 0.003	0.050 ± 0.012
Hg		<0.01	<0.01	<0.01
As		<0.01	<0.01	<0.01
Se		<0.02	<0.02	<0.02
Mo		<0.02	<0.02	<0.02
Sb		<0.02	<0.02	<0.02
Dissolved organic carbon		6.30 ± 0.57	5.76 ± 0.52	4.76 ± 0.43

**Table 5:** The surface of the samples after the fire resistance test, depending on the method of their production (mold casting and 3D printing) and the composition of the mixture (100% C, 95% C + 5% FA, 95% C + 5% MK). The arrows indicate the cracks on the surface of the samples developed after fire resistance tests.

Sample	Mold Casting	3D Printing
100% C	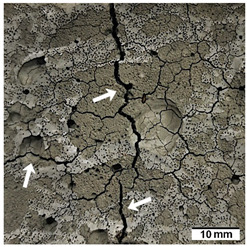	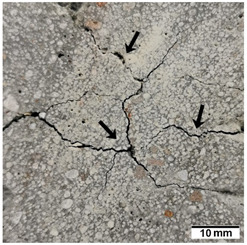
95% C + 5% FA	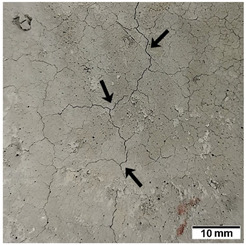	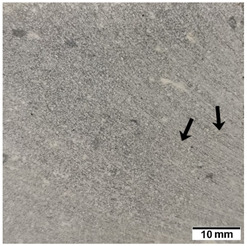
95% C + 5% MK	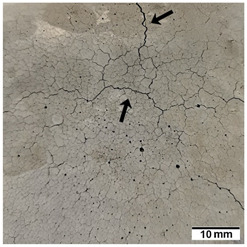	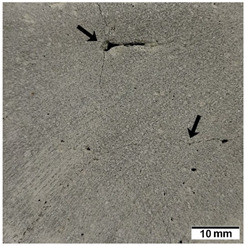

## Data Availability

Not applicable.
